# Supplementary figures and images for: In vitro and in vivo characterization of wild type BMP9 and a non-osteogenic variant in models of pulmonary arterial hypertension
Source: PLoS One. 2025 Jul 28;20(7):e0329089. doi: 10.1371/journal.pone.0329089 (PMC12303310; doi:10.1371/journal.pone.0329089)

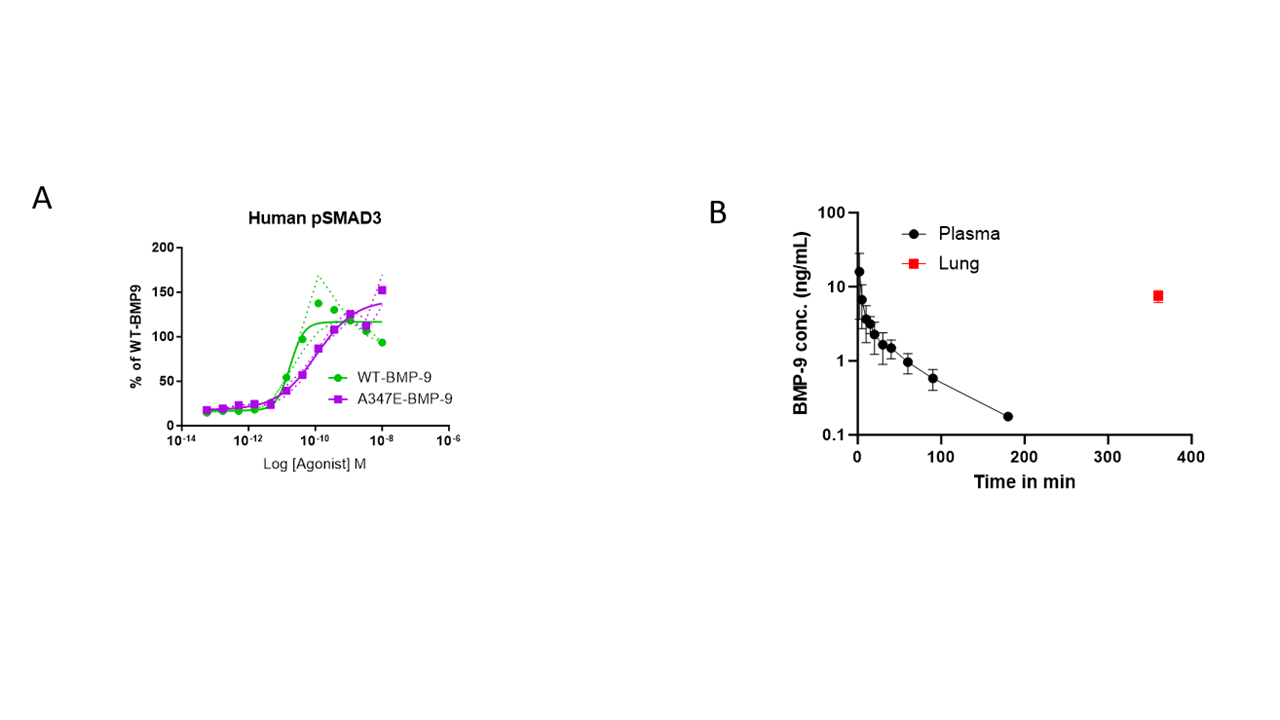

Supplement: S1 Fig — (A) pSMAD3 activation in human endothelial cells in a dose response experiment (0.000169nM-10nM). (B) WT BMP9 (100 µg/kg; IV) plasma- and lung tissue PK evaluation in naïve cynos (n = 3). (TIF) [file pone.0329089.s001.tif]

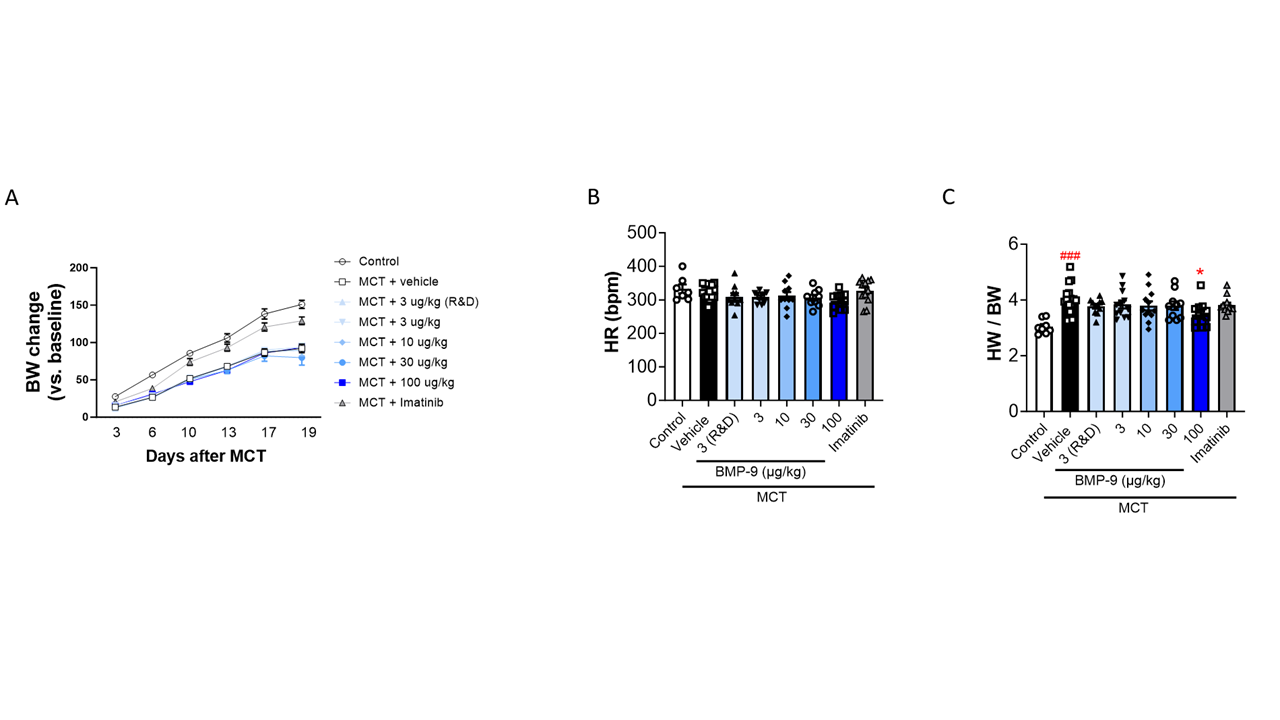

Supplement: S2 Fig — (A) Body weight analysis of vehicle, MCT, WT BMP9 and imatinib rat groups after MCT or vehicle (control) administration (n ≥ 15/group). (B) HR (n ≥ 15/group) and (C) heart weight to body weight (HW/BW) ratio of rats 21 days post MCT induction (n ≥ 15/group). Comparison vs. control: ### p < 0.0049; vs MCT + Vehicle: * p = 0.0183. (TIF) [file pone.0329089.s002.tif]

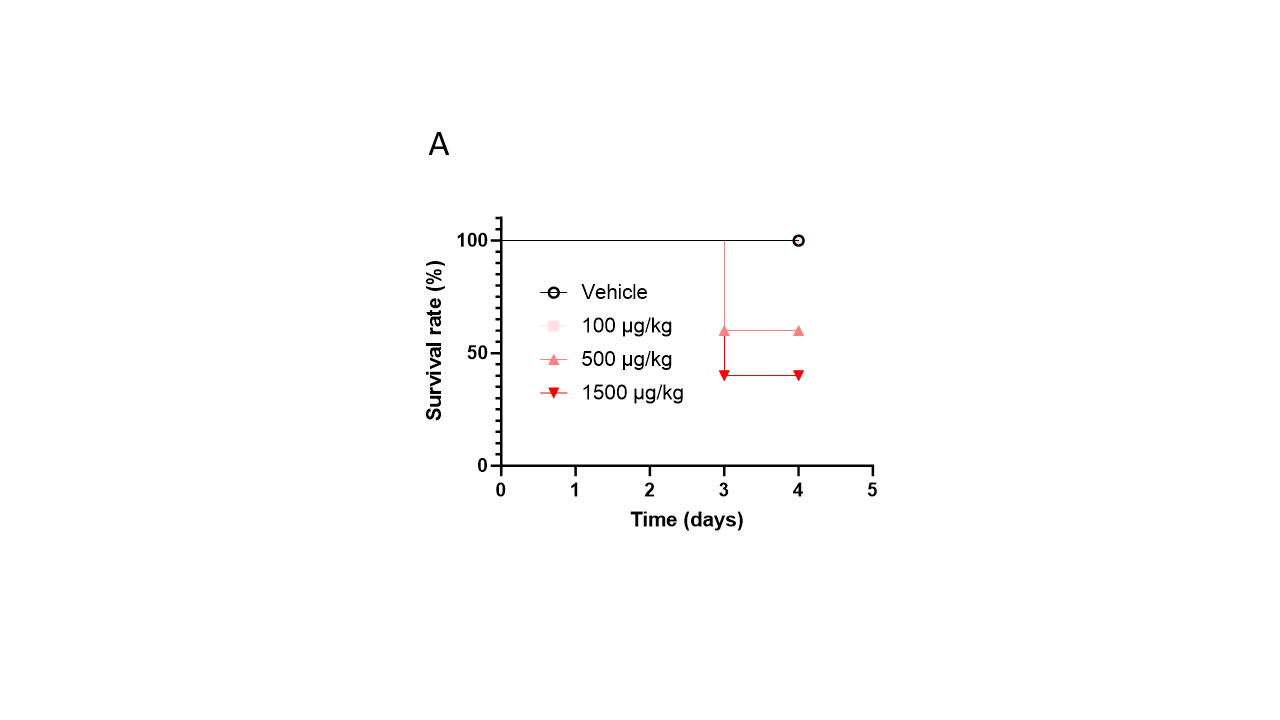

Supplement: S3 Fig — (A) Kaplan-Meier survival curve of naïve rats (n = 5) with continuous IV infusion of WT BMP9 at the indicated dose levels. (TIF) [file pone.0329089.s003.tif]

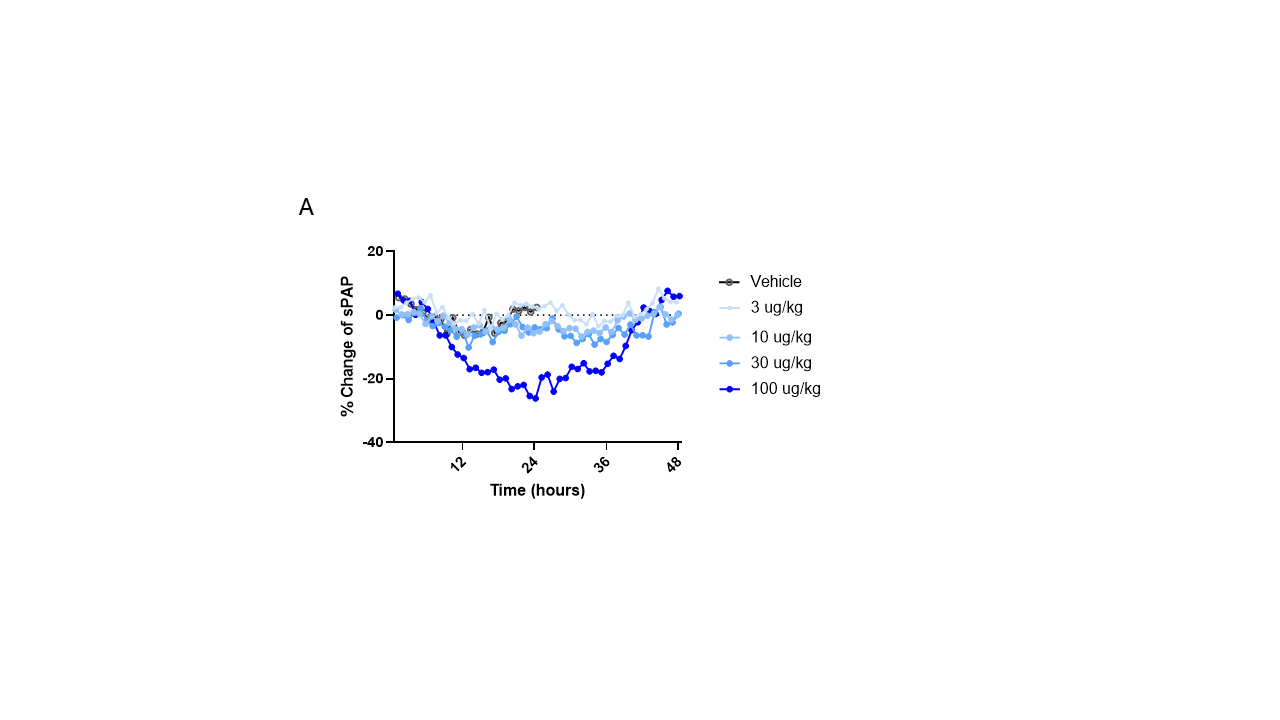

Supplement: S4 Fig — (A) Continuous hemodynamic measurement of sPAP over 48h after a single SC dose of WT BMP9 (3–100 µg/kg) in Su/Hx rats (n = 6). (TIF) [file pone.0329089.s004.tif]
